# Recurrent noncirrhotic hyperammonemia causing acute metabolic encephalopathy in a patient with a continent ileocecal pouch: a case report

**DOI:** 10.1186/s13256-021-02842-1

**Published:** 2021-05-22

**Authors:** T. M. Skipina, S. Macbeth, E. L. Cummer, O. L. Wells, S. Kalathoor

**Affiliations:** Department of Internal Medicine, Wake Forest Baptist Medical Center, 1 Medical Center Boulevard, Winston-Salem, NC USA

**Keywords:** Noncirrhotic hyperammonemia, Urinary diversion, Encephalopathy, Metabolic acidosis, Case report

## Abstract

**Introduction:**

Acute encephalopathy, while a common presentation in the emergency department, is typically caused by a variety of metabolic, vascular, infectious, structural, or psychiatric etiologies. Among metabolic causes, hyperammonemia is relatively common and typically occurs in the setting of cirrhosis or liver dysfunction. However, noncirrhotic hyperammonemia is a rare occurrence and poses unique challenges for clinicians.

**Case presentation:**

Here we report a rare case of a 50-year-old Caucasian female with history of bladder cancer status post chemotherapy, radical cystectomy, and ileocecal diversion who presented to the emergency department with severe altered mental status, combativeness, and a 3-day history of decreased urine output. Her laboratory tests were notable for hyperammonemia up to 289 μmol/L, hypokalemia, and hyperchloremic nonanion gap metabolic acidosis; her liver function tests were normal. Urine cultures were positive for *Enterococcus faecium*. Computed tomography imaging showed an intact ileoceal urinary diversion with chronic ileolithiasis. Upon administration of appropriate antibiotics, lactulose, and potassium citrate, she experienced rapid resolution of her encephalopathy and a significant reduction in hyperammonemia. Her hyperchloremic metabolic acidosis persisted, but her hypokalemia had resolved.

**Conclusion:**

This case is an example of one of the unique consequences of urinary diversions. Urothelial tissue is typically impermeable to urinary solutes. However, when bowel segments are used, abnormal absorption of solutes occurs, including exchange of urinary chloride for serum bicarbonate, leading to a persistent hyperchloremic nonanion gap metabolic acidosis. In addition, overproduction of ammonia from urea-producing organisms can lead to abnormal absorption into the blood and subsequent oversaturation of hepatic metabolic capacity with consequent hyperammonemic encephalopathy. Although this is a rare case, prompt identification and treatment of these metabolic abnormalities is critical to prevent severe central nervous system complications such as altered mental status, coma, and even death in patients with urinary diversions.

## Introduction

Acute encephalopathy is a common presentation in any emergency department. The differential is broad and includes metabolic, vascular, infectious, structural, psychiatric, and medication/substance-induced etiologies.

Among metabolic causes, hyperammonemia is a frequent source of altered mental status and is typically related to cirrhosis or liver dysfunction. However, several nonhepatic etiologies of hyperammonemic encephalopathy have been described in the literature and include urinary tract infections (UTI) with urea-splitting bacteria [1–7], hemato-oncologic disorders [5], organ transplantation [5, 8], ureteral diversions [5, 9, 10], portosystemic shunts [11, 12], medications [5, 13–15], and urea-cycle disorders [16].

Here we report a case of fulminant noncirrhotic hyperammonemic encephalopathy secondary to a UTI in a patient with a continent ileocecal pouch (Indiana pouch) and chronic ileolithiasis.

## Case presentation

A 50-year-old Caucasian female with a history of pulmonary embolism, morbid obesity [body mass index (BMI) 48], chronic abdominal pain, and high grade papillary carcinoma of the bladder (T3bN1) status post chemotherapy, radical cystectomy, and Indiana pouch creation presented to the emergency department with altered mental status and a 3-day history of decreased urine output, foul-smelling urine, nausea, vomiting, and anorexia. She had been hospitalized multiple times in the past for urosepsis, hypokalemia, enterocutaneous fistula, abdominal wall abscesses, and cellulitis. She was most recently hospitalized 3 months prior for altered mental status and was successfully treated with piperacillin–tazobactam that was deescalated to oral ciprofloxacin after a urine culture grew pansensitive *Escherichia coli* and *Proteus vulgaris*. Notably, she performs in-and-out catheterization through her umbilicus several times per day to drain urine from the Indiana pouch.

On physical examination, the patient was belligerent and oriented only to place and person. Neurological examination was grossly normal by observation, but limited by severe patient combativeness. The patient was swinging wildly at staff and screaming during intake interview and examination. History obtained from her mother revealed that at baseline she was a pleasant and well-adjusted individual. Her abdomen was tender to palpation, but without distention or rigidity. A large midline scar was present without erythema or purulence.

Laboratory studies demonstrated hyperammonemia (289 μmol/L), hypokalemia (2.8 mmol/L), hyperchloremia (115 mmol/L), nonanion gap metabolic acidosis, normal liver function studies, mildly elevated creatinine (1.36 mg/dL), chronic leukopenia (2.8 × 1000/mm^3^), and thrombocytopenia (71 × 1000/mm^3^). Urinalysis showed increased pH (8.5) and pyuria with many bacteria. Urine cultures grew 10,000 to 50,000 colony forming units (CFU)/mL *Enterococcus faecium*. Blood cultures were negative. Arterial blood gas was unable to be obtained owing to her mental status.

**Fig. 1:**
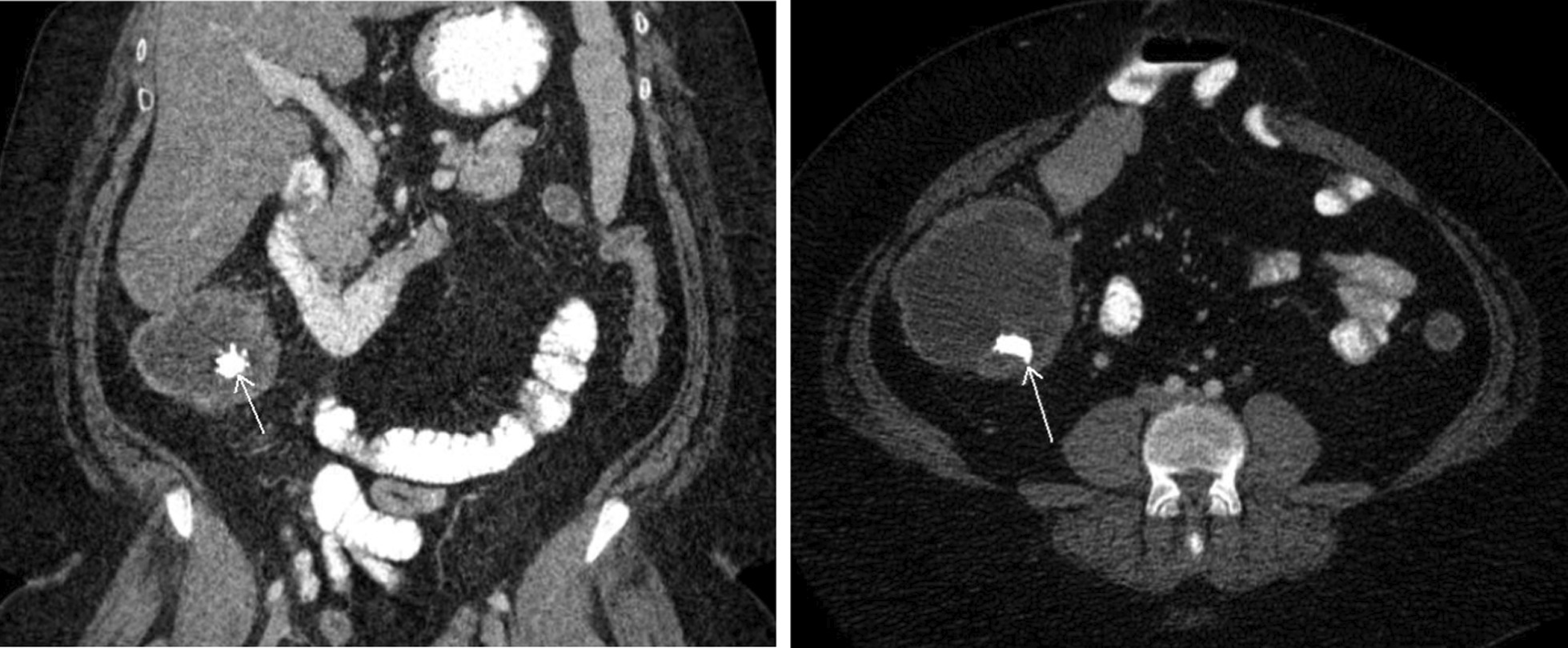
CT of the abdomen and pelvis with contrast. CT scan of the abdomen and pelvis showing postsurgical changes of radical cystectomy with ileal diversion in the right lower quadrant. Ileolithiasis is present within the Indiana pouch (arrow). There is no hydroureteronephrosis. No other acute abnormality is present in the abdomen or pelvis

Computed tomography (CT) imaging showed postsurgical changes of radical cystectomy with ileocecal diversion in the right lower quadrant; there was also ileolithiasis present (Fig. 1). CT of the head was unrevealing.

The patient was initially admitted to the general medicine service, but because of significantly altered mental status and combativeness with concern for airway protection, the patient was ultimately transferred to the medical intensive care unit (ICU) for continued management. Of note, she had presented 3 months prior with similar symptoms that resolved quickly with a course of piperacillin–tazobactam with transition to oral ciprofloxacin. Because of this, she was empirically started on piperacillin–tazobactam, but was changed to daptomycin once urine cultures showed *Enterococcus* growth, and ultimately transitioned to vancomycin thereafter. She was treated with antibiotics for a total of 5 days. Her hyperammonemia was treated with lactulose, and her hypokalemia and nonanion gap metabolic acidosis were treated with potassium citrate.

Ultimately, she returned to her baseline mental status within 3 days and was discharged on lactulose and potassium citrate. Her discharge laboratories tests were notable for an ammonia level of 108 μmol/L and persistent hyperchloremic metabolic acidosis (Table 1).

**Table 1:** Comparison of chemistries on admission and at discharge

Chemistry	On admission	At discharge
Na (mmol/L)	144	138
K (mmol/L)	2.8	3.7
Cl (mmol/L)	115	110
CO_2_ (mmol/L)	18	18
BUN (mg/dL)	20	7
Cr (mg/dL)	1.36	0.93
Anion gap (mmol/L)	11	10
Total bilirubin (mg/dL)	0.5	0.4
AST (IU/L)	27	17
ALT (IU/L)	31	21
Ammonia (μmol/L)	289	108

## Discussion

Noncirrhotic hyperammonemia can be caused by a multitude of factors. Endogenous ammonia, a breakdown product of protein, is primarily processed in the liver. It reacts with bicarbonate and *N*-acetylglutamic acid in the mitochondria to enter the urea cycle. After a multistep process, nontoxic urea is formed in the cytosol and subsequently transported to the kidneys to be excreted in the urine [17].

In general, noncirrhotic or nonhepatic hyperammonemia can be divided into two etiological groups as described by Laish *et al.*, [5]: increased ammonia production or decreased ammonia clearance (Table 2).

**Table 2:** Typical etiologies of nonhepatic hyperammonemia

Increased ammonia production	Decreased ammonia clearance
Infections by urea-producing bacteria: *Proteus mirablis, Klebsiella species, Escherichia coli, Morganella morganii, Providencia rettgeri, diphtheroids, Mycobacterium genavense*, herpes simplex	Ureterosigmoidostomy
Hematooncological disorders: multiple myeloma, chemotherapy for acute leukemia, bone marrow transplantation, 5-fluorouracil	Portosystemic shunts; congenital intrahepatic and extrahepatic
Organ transplantation	Drug-induced: valproic acid, glycine, carbamazepine, ribavirin, sulfadiazine with pyrimethamine, salicylate
Protein load and increased catabolism: severe exercise, seizures, starvation or trauma, total parenteral nutrition, gastrointestinal bleeding, steroid use	Inborn errors of metabolism: urea-cycle disorders, defects in β-oxidation of fatty acids, organic acidemias, disorders of pyruvate metabolism

In our patient, the most likely precipitant of her hyperammonemia was increased ammonia production from a urinary tract infection in the setting of chronic ileolithiasis. She grew *E. faecium* in her urine, and although this organism is not classically known for being a urea producer, there has been a case reported in the literature describing recurrent noncirrhotic hyperammonemia in a patient with urine cultures growing *E. faecalis* [18]. In concordance with this case, there is a possibility that our patient’s strain of *E. faecium* acquired urease-producing capabilities.

The patient has a remote history of malignancy with chemotherapy treatment, but no history of any hematologic disorder or recent chemotherapeutics, making this etiology much less likely. In addition, she has no history of organ transplantation. There were no obvious signs of seizures, starvation, trauma, gastrointestinal bleeding, or any reports of steroid use. Per chart documentation and discussion with family, she had no history of ethanol abuse or liver disease, but has had a history of incidental hepatomegaly on CT. The patient did not appear to be in a prolonged catabolic or cachectic state; however, she did have mildly decreased oral intake in the days leading up to her acute presentation.

Regarding etiologies of decreased ammonia clearance, our patient did not have a ureterosigmoidostomy but had a different configuration of urinary diversion in the form of an ileal neobladder. Hyperammonemia in the presence of bowel segments used as a bladder is not surprising given the fact that the bowel retains its absorptive and secretory capabilities. Urothelial tissue at baseline is a highly impermeable barrier to urinary solutes; urine tends to have a low sodium content and high potassium content and is typically acidic with low concentrations of bicarbonate [19]. Urinary ammonium is created by the kidney in an effort to buffer urinary acidity and is instead reabsorbed by the bowel, where it travels to the liver to be metabolized by the urea cycle [19]. Ammonia is thereby absorbed, resulting in oversaturation of hepatic metabolic capacity and consequential hyperammonemia in the setting of other precipitating factors such as urea-splitting organisms and hepatic or renal insufficiency [19, 20]. Notably, these findings are more commonly reported in patients with ureterosigmoidostomy and not ileal diversion, which suggests that this case is an anomaly among an already rare subset of nonhepatic hyperammonemia related disorders.

Hyperammonemia can also result from decreased absorption of bile salts and interruption of the enterohepatic cycle. The ileum is a site of bile salt and fat absorption; however, when this is removed and used as a urinary diversion, buildup of these contents occurs within the small bowel. This imbalance can lead to small intestine bacterial overgrowth (SIBO), and decreased absorption of alkaline contents leading to acidosis and dehydration [7]. In addition, it is well known that patients with urinary diversion are more prone to dehydration [19, 21]. Given that the ileocecal valve serves as one of the protective factors for SIBO and that our patient had it manipulated and reconstructed, SIBO could have been contributory or precipitative to her presentation [22].

In addition to hyperammonemia, our patient also had hyperchloremic metabolic acidosis, which is a well-documented consequence of urinary diversion using bowel segments [19]. Ileal tissue contains antiporters that exchange ions in equimolar doses; in the setting of urine exposure, urinary chloride is absorbed in exchange for bicarbonate. Since urine typically has little to no bicarbonate and a moderate concentration of chloride, bicarbonate is transported into the urine in exchange for chloride, resulting in the observed hyperchloremic metabolic acidosis [19, 20, 23].

Given that the administration of lactulose and the initiation of antibiotics were essentially simultaneous in our patient, we are unable to differentiate which therapy played a more predominant role in improving her hyperammonemia and neuropsychiatric symptoms. While the mechanism of action of lactulose is still debated in the literature, it has long been hypothesized that lactulose in the gut causes a proliferation of non-urease producing microorganisms (such as *Lactobacilli* and *Bifidobacteria*), which then results in intraluminal lactate and acetate production causing a decrease in the pH of the colon [24, 25]. The acidification of the colon causes NH_3_ (ammonia) to be protonated to NH_4_^+^ (ammonium), and thus, ammonia can no longer diffuse into the bloodstream or move across the blood–brain barrier when it takes the form of ammonium [24, 26]. Additionally, a cathartic effect occurs causing NH_3_ to be removed from the bloodstream, diffused into the gut where NH_4_^+^ is, and then generated and excreted from the body, resulting in a reduction in ammonia levels. Importantly, cerebral edema with subsequent brain herniation is the feared consequence of elevated ammonia levels in both cirrhotic and noncirrhotic patients. This complication develops when ammonia and glutamate are converted into glutamine by glutamine synthetase, which is present in astrocytes [24, 27]. Glutamine behaves as an osmolyte causing astrocytic dysfunction, leading to the development of cerebral edema, which clinically manifests as altered mental status, coma, or death in extreme cases [24, 27]. It is important to note that ammonia levels do not always correlate with encephalopathy in cirrhotic patients [28]; however, we believe that hyperammonemia is the most likely cause of our patient’s altered mental status, given that this was her only severe metabolic abnormality and there was a temporal association between resolution of her encephalopathy and reduction of her hyperammonemia on two different occasions.

Ultimately, our patient’s hyperammonemia was presumed to be from resorption of urinary solutes in the setting of ileal urinary diversion precipitated by a UTI. In the setting of chronic ileolithiasis, this could serve as a nidus for recurrent UTI and subsequent precipitant hyperammonemia. After treatment with antibiotics and lactulose, the patient’s mental status and hyperammonemia improved, and she was subsequently discharged on daily lactulose and potassium citrate for her hyperammonemia, hypokalemia, and metabolic acidosis. If she continues to have recurrences in the future, there is a case report of a patient with recurrent hyperammonemic encephalopathy after a ureterosigmoidostomy who experienced complete resolution of hyperammonemia with diet involving complete restriction of animal proteins and only plant-based protein supplementation, so this could be a potential therapeutic intervention [23]. In addition, stone removal or prophylactic antibiotics could be further interventions should she fail to improve with conservative therapies.

## Conclusion

This case is an example of one of the unique consequences of urinary diversions. Urothelial tissue is typically impermeable to urinary solutes. However, when bowel segments are used, abnormal absorption of solutes occurs, including exchange of urinary chloride for serum bicarbonate, leading to persistent hyperchloremic nonanion gap metabolic acidosis. In addition, overproduction of ammonia from urea-producing organisms can lead to abnormal absorption into the blood and subsequent oversaturation of hepatic metabolic capacity with consequent hyperammonemic encephalopathy. Although this is a rare case, prompt identification and treatment of these metabolic abnormalities is critical to prevent severe central nervous system complications such as altered mental status, coma, and even death in patients with urinary diversions.

## Data Availability

Not applicable.

## References

[1] Triplett KE, Murray R, Anstey M. Multifactorial non-cirrhotic hyperammonaemic encephalopathy. BMJ Case Rep. 2018. 10.1136/bcr-2017-223245. PMC5847978 29523611

[2] Hassan AAI, Ibrahim W, Subahi A, Mohamed A. “All that glitters is not gold”: when hyperammonaemia is not from hepatic aetiology. BMJ Case Rep. 2017. 10.1136/bcr-2017-219441. PMC5753707 28536219

[3] Pallavi R, Matejak-Popis B. Hyperammonemia: a silent killer. Am J Ther. 2016;23(2):e591-593. 10.1097/MJT.0000000000000023 24413369

[4] Kenzaka T, Kato K, Kitao A,* et al*. Hyperammonemia in urinary tract infections. PLoS ONE. 2015;10(8):e0136220. 10.1371/journal.pone.0136220 PMC4546155 26292215

[5] Laish I, Ben AZ. Noncirrhotic hyperammonaemic encephalopathy. Liver Int. 2011;31(9):1259–70. 10.1111/j.1478-3231.2011.02550.x 21745294

[6] LaBuzetta JN, Yao JZ, Bourque DL, Zivin J. Adult nonhepatic hyperammonemia: a case report and differential diagnosis. Am J Med. 2010;123(10):885–91. 10.1016/j.amjmed.2010.02.029 20920686

[7] Van der Aa F, Joniau S, Van Den Branden M, Van Poppel H. Metabolic changes after urinary diversion. Adv Urol. 2011;2011:764325. 10.1155/2011/764325 PMC3113422 21687576

[8] Li GZ, Tio MC, Pak LM,* et al*. Noncirrhotic hyperammonemia after deceased donor kidney transplantation: a case report. Am J Transplant. 2019;19:3197–201. 10.1111/ajt.15545 PMC6864227 31347272

[9] Perez-Fidalgo JA, Chirivella Gonzalez I, Gunthner S, Cervera Miguel JI, March Villalba JA, Cervantes Ruiperez A. Hyperammonaemic encephalopathy, possible complication after urinary diversion in radical cystectomy. Review of the literature with regard to a clinical case. Actas Urol Esp. 2007;31(4):394–9. 10.1016/s0210-4806(07)73654-9 17633926

[10] Weiss N, Levi C, Hussenet C,* et al*. A urinary cause of coma. J Neurol. 2011;258(5):941–3. 10.1007/s00415-010-5850-7 21136271

[11] Rogal SS, Hu A, Bandi R, Shaikh O. Novel therapy for non-cirrhotic hyperammonemia due to a spontaneous splenorenal shunt. World J Gastroenterol. 2014;20(25):8288–91. 10.3748/wjg.v20.i25.8288 PMC4081705 25009405

[12] Kasagi Y, Saeki H, Akahoshi T,* et al*. Non-cirrhotic portal-systemic encephalopathy caused by enlargement of a splenorenal shunt after pancreaticoduodenectomy for locally advanced duodenal cancer: report of a case. Surg Today. 2014;44(8):1573–6. 10.1007/s00595-013-0679-1 PMC4097198 23982193

[13] Vivekanandan S, Nayak SD. Valproate-induced hyperammonemic encephalopathy enhanced by topiramate and phenobarbitone: a case report and an update. Ann Indian Acad Neurol. 2010;13(2):145–7. 10.4103/0972-2327.64638 PMC2924516 20814502

[14] Labib PL, Wing S, Bhowmik A. Transient hyperammonaemia in a patient with confusion: challenges with the differential diagnosis. BMJ Case Rep. 2011. 10.1136/bcr.03.2011.3961. PMC3176359 22679184

[15] Dixit S, Namdeo M, Azad S. Valproate induced delirium due to hyperammonemia in a case of acute mania: a diagnostic dilemma. J Clin Diagn Res. 2015;9(4):VD01–2. 10.7860/JCDR/2015/11830.5758 PMC4437142 26023626

[16] Upadhyay R, Bleck TP, Busl KM. Hyperammonemia: what urea-lly need to know: case report of severe noncirrhotic hyperammonemic encephalopathy and review of the literature. Case Rep Med. 2016;2016:8512721. 10.1155/2016/8512721 PMC5050374 27738433

[17] Walker V. Ammonia metabolism and hyperammonemic disorders. Adv Clin Chem. 2014;67:73–150. 10.1016/bs.acc.2014.09.002 25735860

[18] Cordano C, Traverso E, Calabro V,* et al*. Recurring hyperammonemic encephalopathy induced by bacteria usually not producing urease. BMC Res Notes. 2014;7:324. 10.1186/1756-0500-7-324 PMC4057615 24884855

[19] Roth JD, Koch MO. Metabolic and nutritional consequences of urinary diversion using intestinal segments to reconstruct the urinary tract. Urol Clin N Am. 2018;45(1):19–24. 10.1016/j.ucl.2017.09.007 29169447

[20] Moriana M, Martinez-Ibanez J, Civera M, Martinez-Valls JF, Ascaso JF. Hyperammonemic encephalopathy after urinary diversion. Diet therapy. Endocrinol Nutr. 2016;63(6):306–8. 10.1016/j.endonu.2016.03.006 27094153

[21] McDougal WS. Bladder reconstruction following cystectomy by uretero-ileo-colourethrostomy. J Urol. 1986;135(4):698–701. 10.1016/s0022-5347(17)45822-8 3959189

[22] Bures J, Cyrany J, Kohoutova D,* et al*. Small intestinal bacterial overgrowth syndrome. World J Gastroenterol. 2010;16(24):2978–90. 10.3748/wjg.v16.i24.2978 PMC2890937 20572300

[23] Hautmann RE, Miller K, Steiner U, Wenderoth U. The ileal neobladder: 6 years of experience with more than 200 patients. J Urol. 1993;150(1):40–5. 10.1016/s0022-5347(17)35392-2 8510272

[24] Wijdicks EFM. Hepatic encephalopathy. N Engl J Med. 2017;376(2):186. 10.1056/NEJMc1614962 28076712

[25] Wang JY, Bajaj JS, Wang JB,* et al*. Lactulose improves cognition, quality of life, and gut microbiota in minimal hepatic encephalopathy: a multicenter, randomized controlled trial. J Dig Dis. 2019;20(10):547–56. 10.1111/1751-2980.12816 31448533

[26] Ruszkowski J, Witkowski JM. Lactulose: patient- and dose-dependent prebiotic properties in humans. Anaerobe. 2019;59:100–6. 10.1016/j.anaerobe.2019.06.002 31176002

[27] Cudalbu C, Taylor-Robinson SD. Brain edema in chronic hepatic encephalopathy. J Clin Exp Hepatol. 2019;9(3):362–82. 10.1016/j.jceh.2019.02.003 PMC6637228 31360029

[28] Vilstrup H, Amodio P, Bajaj J,* et al*. Hepatic encephalopathy in chronic liver disease: 2014 Practice Guideline by the American Association for the Study of Liver Diseases and the European Association for the Study of the Liver. Hepatology. 2014;60(2):715–35. 10.1002/hep.27210 25042402

